# Co-Density Distribution Maps for Advanced Molecule Colocalization and Co-Distribution Analysis

**DOI:** 10.3390/s21196385

**Published:** 2021-09-24

**Authors:** Ilaria De Santis, Luca Lorenzini, Marzia Moretti, Elisa Martella, Enrico Lucarelli, Laura Calzà, Alessandro Bevilacqua

**Affiliations:** 1 Department of Medical and Surgical Sciences (DIMEC), Alma Mater Studiorum—University of Bologna, I-40138 Bologna, Italy; i.desantis@unibo.it; 2 Interdepartmental Center Alma Mater Research Institute on Global Challenges and Climate Change (Alma Climate), Alma Mater Studiorum—University of Bologna, I-40126 Bologna, Italy; 3 Department of Veterinary Medical Sciences (DIMEVET), Alma Mater Studiorum—University of Bologna, I-40064 Ozzano Emilia, Italy; luca.lorenzini8@unibo.it; 4 Iret Foundation, I-40064 Ozzano Emilia, Italy; marzia.moretti3@unibo.it (M.M.); laura.calza@unibo.it (L.C.); 5 Institute of Organic Synthesis and Photoreactivity (ISOF), National Research Council (CNR), I-40129 Bologna, Italy; elisa.martella@isof.cnr.it; 6 Regenerative Therapies in Oncology, IRCCS Istituto Ortopedico Rizzoli, I-40136 Bologna, Italy; enrico.lucarelli@ior.it; 7 Department of Pharmacy and BioTechnology (FaBiT), Alma Mater Studiorum—University of Bologna, I-40127 Bologna, Italy; 8 Advanced Research Center on Electronic Systems (ARCES) for Information and Communication Technologies “E. De Castro”, Alma Mater Studiorum—University of Bologna, I-40125 Bologna, Italy; 9 Department of Computer Science and Engineering (DISI), Alma Mater Studiorum—University of Bologna, I-40136 Bologna, Italy

**Keywords:** local density, local co-density, co-occurrence, correlation, colocalization quantification, data visualization, fluorescence microscopy, subcellular local image analysis, cancer cell

## Abstract

Cellular and subcellular spatial colocalization of structures and molecules in biological specimens is an important indicator of their co-compartmentalization and interaction. Presently, colocalization in biomedical images is addressed with visual inspection and quantified by co-occurrence and correlation coefficients. However, such measures alone cannot capture the complexity of the interactions, which does not limit itself to signal intensity. On top of the previously developed density distribution maps (DDMs), here, we present a method for advancing current colocalization analysis by introducing co-density distribution maps (cDDMs), which, uniquely, provide information about molecules absolute and relative position and local abundance. We exemplify the benefits of our method by developing cDDMs-integrated pipelines for the analysis of molecules pairs co-distribution in three different real-case image datasets. First, cDDMs are shown to be indicators of colocalization and degree, able to increase the reliability of correlation coefficients currently used to detect the presence of colocalization. In addition, they provide a simultaneously visual and quantitative support, which opens for new investigation paths and biomedical considerations. Finally, thanks to the *coDDMaker* software we developed, cDDMs become an enabling tool for the quasi real time monitoring of experiments and a potential improvement for a large number of biomedical studies.

## 1. Introduction

In a biological context, colocalization is defined as the presence of two or more different molecules residing at the same physical location in a specimen. Subcellular spatial colocalization analysis is fundamental for determining whether molecules are located in sites where they can interact with each other, especially when their reciprocal interaction and reaction cannot be assessed directly. The molecules’ location can be easily and efficiently addressed by confocal fluorescence microscopy [1]: while fluorescent probes allow the selective visualization of specifically marked molecules [2], the confocality of acquisition allows the investigation of their distribution in the whole cellular volume, while reducing the out-of-focus contributions to probe’s signal [3,4] and avoiding image blurring accordingly, which can introduce false positives. A first common method to analyze colocalization of fluorescent signals is image superposition (i.e., merging or, more technically, fusion) for visual inspection [5,6]. However, such a method is subject to perceptive errors and bias [7], cannot discriminate between random and potentially functional colocalization [8] and is poorly quantitative [9]. Accordingly, several methods for quantifying colocalization have been developed through years. A first discrimination occurs between pixel-based and object-based methods [10,11]. As for many other applications, the former is based solely on the intensity information in each pixel, while the latter is based on information from a set of semantically coherent pixels, called the object. Therefore, object-based methods are more appropriate for super-resolution microscopy, which is more suitable for accurately separating interacting molecules in adjacent pixels and discerning objects boundaries [12,13], while the application of pixel-based methods is quite independent of microscopy resolution. Pixel-based methods conventionally regard colocalization as quantifiable by two components [14]: co-occurrence, i.e., the simple spatial overlap of two signals, and correlation, which quantifies the direction and indicate the magnitude of the relation between markers’ signal intensities [13,15]. This approach has given rise to a large number of different correlation coefficients [8,16,17,18,19,20], first of all, Pearson’s [21] and Manders’ [6] coefficients, for their ease of implementation [22] and their capability to provide, respectively, a quantification of correlation and co-occurrence, when used in pair [6,7,13]. The derived forms of these coefficients have been progressively introduced to overcome their main drawbacks, such as noise dependency [23], lack of linearity [14] and absence of spatial informativeness [20]. However, their adoption is still limited by their shared inadequacy to provide an intuitive and effective representation of colocalization that could really help researchers in the biological interpretation of results. In addition, none of them can provide information about the stoichiometry of colocalization [3], which is still approximated from the pixel intensities scatterplot as the slope of the fitting line assuming, a priori, a linear relation between the two signals intensities [8,24,25].

All methods exploiting pixel intensities neglect information regarding pixel interconnections that, if considered, could permit the enforcement of colocalization information. In fact, co-localized pixels, by definition, must appear with the same connecting pattern in both channels. Based on this assumption, we developed the concept of density distribution maps (DDMs) [26], which qualitatively and quantitatively describe the subcellular molecules’ absolute and relative locations. As a natural extension, here, we introduce the co-density distribution map (cDDM), a novel tool to automatedly and quantitatively improve colocalization analysis in biomedical images by firstly introducing information about molecules local density and co-density. Consequently, cDDMs borrow all of the advantages of DDMs, including the capability to increase the confidence of colocalization when this is not achievable by increasing the image resolution; the capability to speed-up routine, large-scale and follow-up experiments; and the applicability to any resolution study. Working on densities, cDDMs introduce an additional constraint that makes the overall colocalization assessment more reliable, becoming a tool for the refinement of correlation coefficients computation, when these coefficients are chosen as quantifiers of colocalization. In practice, cDDMs provide a more reliable indication than intensity alone about the location and extent of colocalization (that is by definition a spatial information, as local density). In addition, being representative of markers’ local co-density, cDDMs offer a visual support preserving the spatial information and making the biological interpretation of results easier. After presenting the cDDMs’ creation method and discussing its main implications, here, we exemplify the effectiveness of cDDMs through their application to two more real image datasets acquired by fluorescence microscopy, which prove how cDDMs can advance the actual colocalization analysis framework, provide information about markers’ density and degree of colocalization and, thus, open to the formulation of new biological considerations. Finally, we supply an updated version of the software program *DDMaker* [26], *coDDMaker* and its Graphical User Interface (GUI), to support researchers in building and analyzing the DDMs and cDDMs for their own experiments.

## 2. Materials and Methods

Three datasets are used to exemplify cDDMs’ benefits to biomedical colocalization and co-distribution studies: (1) the SYP-VGLUT1 dataset is used to present cDDM utilization and main implications (results for the SYP-VGLUT1 dataset are also reported in the Supplementary Table S1); (2) the Lamp-1-Ce6 dataset is used to present a case of limited colocalization between differently dense markers, where the analysis is complemented by novel information from the cDDM, including indication on the degree of colocalization; (3) the NF200-FM dataset is used to present a case of cDDMs application at tissue level, where local co-density numerical and spatial information also permits new biological considerations about sample’s heterogeneity.

### 2.1. Sample Preparation and Image Acquisition

(1) The 12 bit range images from rat brain immunostained for Synaptophysin (SYP, λ_EX_ = 488 nm, λ_EM_ = 525 nm) and vesicular glutamate transporter 1 (VGLUT1, λ_EX_ = 561 nm, λ_EM_ = 595 nm), as described in [27], were sequentially acquired with a Nikon Ti-E A1R laser confocal fluorescence microscope (Nikon, Tokyo, Japan), equipped with a Plan Apo 60x/1.4 objective at a resolution of 512 × 512 × 9 pixels with a pixel size (XYZ) of 0.1 × 0.1 × 0.25 µm^3^ (Pinhole size = 39.59 µm). (2) The 12 bit range images of human osteosarcoma MG-63 cells exposed to Keratin-based nanoparticles (PTX-Ce6@ker_ag_, λ_EX_ = 649 nm, λ_EM_ = 700 nm) were sequentially acquired with a confocal fluorescence laser scanning microscope Ti-E A1R (Nikon, Amsterdam, Netherlands), equipped with a 60×/NA 1.4 oil Plan-Fluo at a resolution of 1024 × 1024 × 19 pixels with a pixel size (XYZ) of 0.2 × 0.2 × 0.25 µm^3^ (Pinhole size = 24.27 µm). MG-63 cells were indirectly immunostained against the Lysosomal-associated membrane protein 1 (Lamp-1, λ_EX_ = 563 nm, λ_EM_ = 595 nm) as described in [28]. 3) The 8 bit range images from rat spinal cord immunostained for neurofilaments (NFs, primary antibody: mouse anti-NF200, 1:800, Sigma Aldrich Saint Louis, MO; secondary antibody: Rhodamine Red™-X, 1:100, Jackson Immuno Research, Cambridgeshire, UK, λ_EX_ = 570 nm, λ_EM_ = 590 nm) and stained for myelin with FITC-Fluoromyelin™ (FM, Thermo Fisher, λ_EX_ = 479 nm, λ_EM_ = 598 nm) were acquired with a Nikon Eclipse E600 (Q Imaging, Surrey, BC, Canada), equipped with a Plan Apo 10x/0.4 objective and Q Imaging RETIGA-2000RV camera. For each sample, 10 images were acquired and stitched into a single mosaic (resolution: 3532 × 2384 pixels, pixel size: 0.74 × 0.74 µm^2^) with Photoshop (Adobe Suite, release 22.4.2).

### 2.2. Image Segmentation

All the following procedures are implemented in MATLAB^®^ (R2019a v.9.7.0, The MathWorks, Natick, MA, USA). SYP and VGLUT1 signals are segmented by Isodata thresholding. Lamp-1 and Ce6 signals and NF200 and FM signals are segmented by Otsu method.

### 2.3. Local Distribution and Co-Distribution Analysis, DDM and cDDM

**Figure 1:**
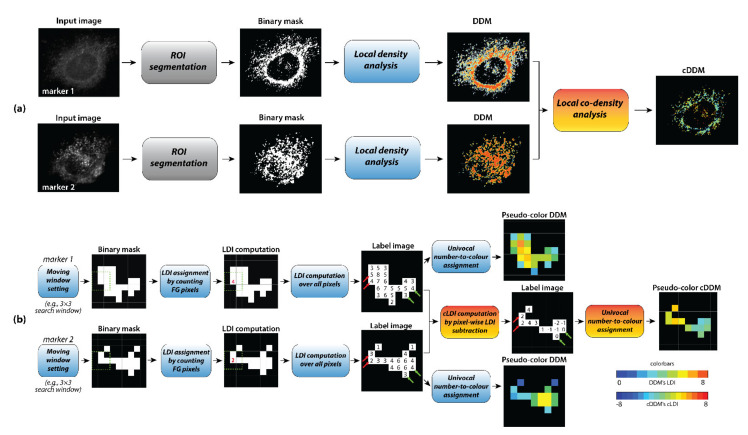
Flowchart of cDDM creation pipeline for a couple of markers. (**a**) The acquired images are segmented in binary masks and their pixel connectivity separately explored by local density analysis for the two pseudo-color DDMs building. Then, the cDDM is built through local co-density analysis, by comparing the single markers DDMs pixelwise. (**b**) Details of local density (blue boxes) and co-density (orange boxes) analyses: after setting the search (moving) windows size, each foreground (FG) pixel of each binary mask is assigned a number representing the amount of FG pixels in its locality, this constituting the input to build the pseudo-color DDM (here shown with no “saturated” densities). Then, the local co-density analysis is performed by pixelwise subtraction of the two DDMs.

Starting from pairs of input grey level images, the cDDM is computed from single DDMs (Figure 1a).

As schematized in Figure 1b, distribution analysis is performed by firstly computing the local density indices (LDIs) and DDM of each imaged marker, as described in [26], after setting the search (moving) Windows Size (WS), which can differ for the two markers. Then, for each pair of markers, the co-distribution analysis is performed by computing the co-density distribution map (cDDM), by subtracting the two markers’ DDMs pixelwise. Consequently, the resulting cDDM’s values (i.e., local co-density indices, cLDIs) can be only computed inside the markers’ co-occurrence region, resulting from ANDing the two markers’ masks and can range from −(WS^2^ − 1) to +(WS^2^ − 1). Different LDI couples can result in the same cLDI (Figure 1b, red and green arrows). Negative cLDI values indicate pixels where the first marker signal is locally denser than the second one, the opposite holds for positive values. A cLDI equal to zero indicates pixels where the two markers are equally dense, hence defining the equi-density region, where the signals are in a 1:1 ratio. However, non-zero cLDIs cannot be considered indicators of a specific ratio, but rather, of a specific difference in the markers’ abundance that is, by definition, a more correct indication of the degree of colocalization than of pixel intensities correlation. Finally, mapping cLDIs back to the image domain in pseudo-colors also allows us to gain information about the markers’ spatial co-distribution.

### 2.4. Pixel Density as a Measure of Colocalization

An established requirement for signals colocalization is their co-occurrence. Co-occurring pixels can either be isolated (i.e., they have no neighboring pixels) or not. If we assume that isolated pixels as the result of spurious co-occurrences, colocalization is, hence, defined by the presence of at least two adjacent co-occurring pixels. This means that colocalization presents itself in patterns, in their turn defined by connections between pixels. As a consequence, there is the necessity to also quantify colocalization with a measure of pixel connectivity (i.e., our local co-density), rather than using an intensity-based measure alone. Assuming that the objects of interest to be imaged are larger than single pixels, the 3 × 3 search window (i.e., WS = 3) is the smallest window to analyze pixel connectivities and, hence, local densities. Such an assumption is fundamental to determine whether the local co-density information carried by cDDMs also brings information about objects colocalization. Indeed, when imaging single-pixel objects, cLDI cannot be indicative of colocalization, being unable to discriminate between a real overlap and a close proximity, since non-overlapping single-pixel objects can fall within a single pixel. In such cases, more information about colocalization can be drawn from pixel-based correlation coefficients, under the assumption of proportionality between marker intensity and molecule number. Such an assumption is not exploited in our method, which relies on a more straightforward measure of the marked objects abundance based on local density.

Hence, co-density is a measure of colocalization when the search window has a size that is, at most, the same as that of the imaged objects. In such cases, a cLDI value of zero indicates the presence of co-occurring and co-dense objects, thus identifying those pixels where two signals colocalize not only because they co-occur (and perhaps correlate), but also because they do it by sharing the same pattern density.

### 2.5. Colocalization Analysis

In this work, we implement a colocalization analysis framework according to the most common methods in the biomedical literature. Specifically, we quantify the signals overlap by Manders’ coefficients MOC, M_1_ and M_2_, and signals correlation by Pearson’s (ρ) and Spearman’s (ρ_s_) [29] coefficients. Of note, MOC’s informativeness as a co-occurrence estimator is actually an ongoing topic of discussion [4,30,31,32,33] and the MOC values reported hereafter should be carefully interpreted accordingly. The formulae and description for the mentioned coefficients can be found in Appendix A. In addition, we also evaluate:

- The markers overlap region through our co-occurrence maps (cOMs) built on top of segmented signals, highlighting in four different pseudo-colors the pixels where: (1) both markers are absent, (2) only the first marker is present, (3) only the second marker is present and (4) both markers are present (co-occurrence region).
- The local density and co-density of marked structures, by DDMs and cDDMs computation and analysis.

### 2.6. Assessment of Results

We first verify the appropriateness of cLDI as a colocalization indicator by assessing the degree of an order relation between cLDI and correlation coefficient values. Hence, we apply a cLDI-based refinement of classical coefficients computation, which consists in restricting its domain from the co-occurrence region to the equi-density region.

For each image, each marker’s signal is binarized in a mask representing its own occurring region. Then, the two masks are ANDed to identify the signals’ co-occurrence region. Finally, the cDDM analyzes the co-occurrence region, restricting it to the co-density region. Correlation (by ρ and ρ_s_) and overlap (by MOC, M_1_ and M_2_) are calculated for both the signals’ intensities (i.e., between the pixel values in the two markers’ images) and the signals’ local density (i.e., between the pixel values in the two markers’ DDMs) to assess to what extent density and intensity are comparable descriptors of colocalization. The signals’ intensity correlation (and MOC) is calculated in three increasingly narrowed domains: the entire image, the co-occurrence region and the co-density region. As expected, the first narrowing, from the entire image to the co-occurrence region, always decreases the correlation coefficients value, excluding the random colocalization of the background (data not shown). M_1_ and M_2_ coefficients are calculated for signals’ intensity with respect to both the co-occurrence and the co-density regions, according to equations (3) and (4) of Appendix A, where the “colocalizing” pixels at the numerators are the co-occurring and the co-dense pixels, respectively. The signals’ density correlation and co-occurrence are calculated only for the co-occurrence region. Indeed, density computation is theoretically impossible before the co-occurrence region definition, whilst inside the co-density region, the coefficients values would be biased by the density-based nature of the refinement itself (i.e., all coefficients value would be set to 1).

In addition, we also compare our cDDM-based method to binary erosion for the restriction of the co-occurrence region, using *4-connected* and *8*(*full)-connected* kernels. However, considering full-connection for comparison is probably fairer, since cDDMs also explore full connectivity. The comparison regards the number of pixels and objects in the masks, as well as the correlation coefficients (ρ and ρ_s_) value, before and after pixel removing by erosion and pixel selection by cDDMs.

More benefits and the effectiveness of cDDMs are then discussed in three examples.

## 3. Results and Discussion

### 3.1. Functional Implication of cDDMs

**Figure 2:**
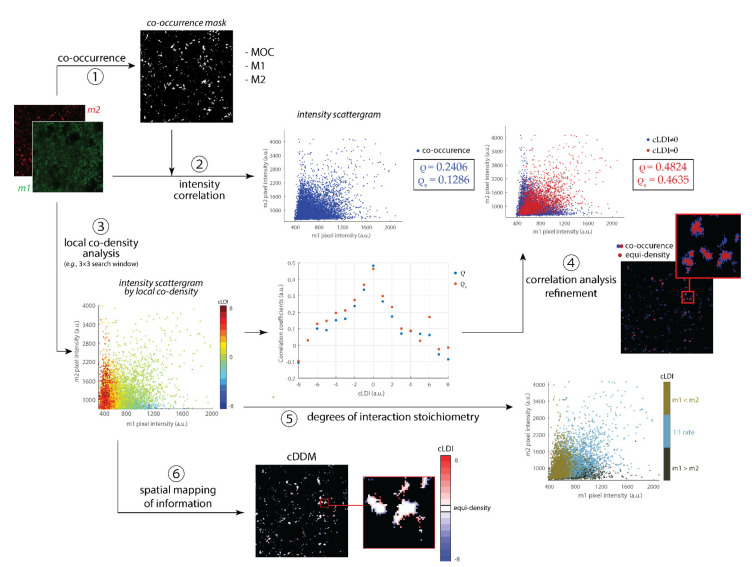
Functional implications of coDDM. Starting from a couple of marked images (m1 and m2), colocalization is usually quantified as a combination of markers overlap (by co-occurrence mask and Manders’ MOC, M_1_ and M_2_ coefficients computation, ①) and intensity correlation (primarily by ρ and ρ_s_ correlation coefficients, ②). By cLDIs computation, co-occurrence pixels can be further partitioned by their local co-density and resulting groups visualized in a pseudo-color scattergram (③). When quantifying colocalization through markers’ intensity correlation, the analysis specificity can be increased by narrowing the computational domain from the co-occurrence to the equi-density region (i.e., made of pixels with cLDI = 0, ④). In addition, being based on density instead of intensity, cLDIs are more appropriate for estimating markers’ relative abundance (⑤). Finally, cDDM permits to preserve the spatiality of original images, additionally coding it with colors for the regional investigation of cLDI distribution (⑥). Details of presented scatterplot data in Supplementary Figure S1A–C.

Colocalization can be defined as the functional and non-spurious co-presence of molecules, most commonly at the single-pixel level. While co-presence can be easily assessed, its functionality must be inferred by other measures, such as signal correlation. However, correlation between coexistent signals does not prove, but only suggests, the presence of colocalization. Such a suggestion can be then corroborated by local co-density analysis that, working locally, improves the information of co-location and, being in an order relation with correlation coefficients, can serve to improve the specificity of colocalization analysis. The main functional implications of cDDMs are schematized in Figure 2.

**Figure 3:**
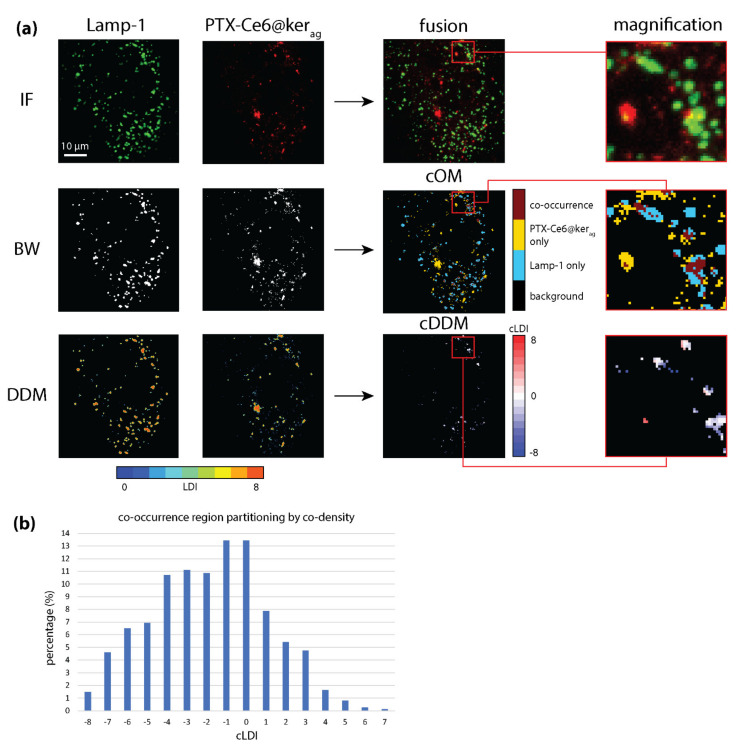
cDDM discloses information about the degree of colocalization. (**a**) Top: Exemplificative immunofluorescence (IF) images of MG-63 cells exposed to PTX-Ce6@ker_ag_ nanoparticles, marked against late endosomes (Lamp-1), with Ce6 (NPs), or both (fusion). Middle: Lamp-1 and Ce6 signals’ binary masks (BW), whose combination produce the co-occurrence map (cOM). Bottom: Lamp-1 and Ce6 DDMs and cDDM. (**b**) Bar graph of co-occurrence region partitioning by co-density, showing a prevalence of negative cLDI values that indicate NPs as generally denser than late endosomes.

**Figure 4:**
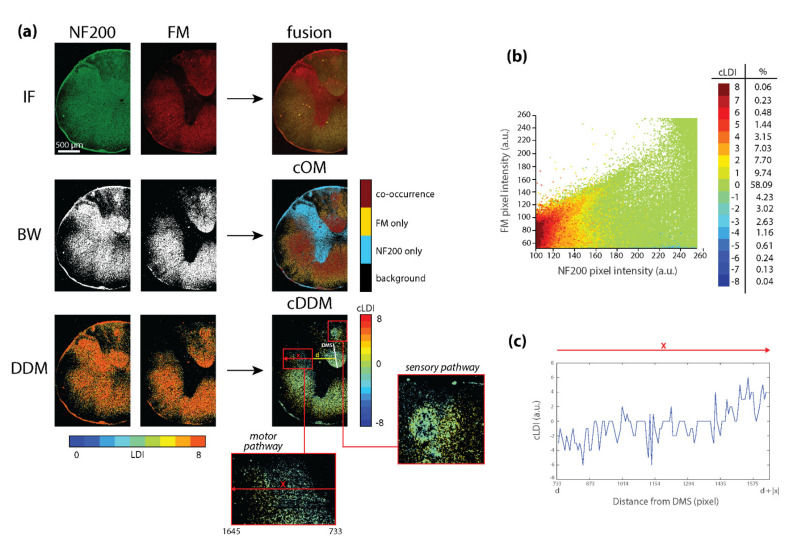
cDDM opens to the formulation of new biological considerations. (**a**) Top: Exemplificative immunofluorescence (IF) images of rats’ spinal cord, marked against the axonal (NF200), the myelin (FM) components of the cord, or both (fusion). Middle: NF200 and FM signal binary masks (BW), whose combination produce the co-occurrence map (cOM). Bottom: NF200 and FM DDMs and cDDM. (**b**) Scattergram of NF200 and FM signals intensity color-coded by cLDI, showing a clear prevalence of equi-density pixels (grey, cLDI *=* 0). (**c**) The line plot reports the cLDI values underlying the horizontal red arrow (x) inside the “motor pathway” magnification. The cLDI medio-lateral distribution is shown in function of the pixel distance (d, yellow line) from the dorsal median sulcus (DMS, white line), highlighting a progressive myelin thinning from spinal cord center to periphery.

Colocalization is usually quantified by markers overlap and intensities in correlation coefficients within the co-occurrence region (Figure 2, ① and ②), defined by the intersection of the two markers (m1, m2) masks. cLDIs computation allows the region to be split into subregions of homogeneous co-density, each one consisting of the set of pixels at which LDI_m1_ − LDI_m2_ = n, where n is a specific cLDI value (Figure 2, ③). If we now compute the correlation coefficients (ρ and ρ_s_) within each cLDI-defined subregion (Figure 2, central scattergram), we can see that correlation between signals intensities increases as cLDI moves from the highest (in absolute terms, i.e., |cLDI| = 8) to the equi-density condition (i.e., cLDI = 0). This proportionality confirms that cLDIs can serve as indicators of colocalization, just as ρ and ρ_s_, at least when they hold. Then, cDDMs can be applied for a density-based refinement of colocalization quantification by correlation coefficients, namely, by restricting their computation from the co-occurrence region to the equi-density one (Figure 2, ④). Apparently, the same restriction of the computational domain could be obtained by a simply binary erosion. However, even under the additional assumption of negligible colocalization at the edge of the co-occurrence region, a refinement by erosion would remove the outer pixels independently of their connection or the presence of colocalization. If this could produce a somewhat lightly divergent set of results when the co-occurrence region is dense (i.e., the edge pixels are a clear minority), the erosion would yield an increasingly invalid outcome as the border indentation of the co-occurrence region increases, or in the presence of small objects. Table 1 reports all of the results, from the initial whole co-occurrence mask to the final masks, achieved by erosions and cDDM, used to assess colocalization. Accordingly, the numbers of edge pixels are complementary (e.g., for NF200-FM the percentage of edge pixels is 34.65).

The co-occurrence region’s border indentation is quantifiable by the number of edge (border) pixels. Therefore, eroding with 4-connectivity makes the effects of indentation decrease from the NF200-FM dataset (35% of co-occurring pixels are on the region’s border) to SYP-VGLUT1 (68%) and Lamp1-Ce6 (88%), which shows the smallest objects. As expected, this trend still holds when eroding by considering full connectivity of pixels, as cDDM does. We can also see that the masks achieved with 4-conn erosion are the widest ones (i.e., having the highest number of pixels), while showing the worst correlations (hence, the worst colocalization performances) over all datasets. This definitely improves with 8-conn, although the mask achieved yields correlation values that still are poor for SYP-VGLUT1 and Lamp1-Ce6. On the contrary, the masks achieved by cDDMs yield the best correlation coefficients, the only mask to bring fair correlations in the two aforementioned datasets. Of course, the best result is achieved with Lamp1-Ce6 because, having small objects, the effect of keeping (co-dense) edge pixels is emphasized. In practice, cDDMs preserve those edge pixels, removed by the erosion without distinction, deserving to be semantically retained instead, since contributing to the measured correlation, independently of their position within the co-occurrence region. Therefore, cDDMs end in preserving a greater number of meaningful pixels and objects than erosion, thus representing a tool for the more precise mapping of stronger colocalization regions.

Let us now deepen the analysis of the results using cDDMs. We have seen that, exploiting pixels’ density, cDDMs can also provide information about the degree of colocalization (Figure 2, ⑤). The markers’ stoichiometric ratio of interaction is sometimes inferred from the slope of the fitting line in the intensity scattergram [22]. However, such an approach riskily depends on the assumption of linearity between the markers’ intensities, that is not the rule when working with biological samples. Instead, cLDI reflects markers’ density and is then, by definition, a more appropriate indicator of the markers’ relative abundance, even when not relying on linearity assumptions.

Although, in the previous case, we used the co-density information at a global level, to compare it to current colocalization methods, we can exploit the locality nature of cDDMs to open for new investigation paths at the regional level (Figure 2, ⑥). Guided, for instance, by anatomical or functional motivations, co-densities distributions can be investigated in specific image subregions or, in the opposite way around, specific co-densities can be addressed one at a time and their distribution singularly investigated at each local level. As attested, especially by the last two datasets, cDDMs can more generally open for the formulation of new biological considerations, as they include spatial quantitative information (neglected by most coefficients), which are also locally computed, to provide a more detailed and comprehensive overview of the investigated system.

Finally, cDDMs borrow all of the advantages of DDMs: first, the capability to provide a more accurate estimation of molecules’ position and an increased robustness to resolution variations based on DDMs’ local density analysis [26]; second, cDDMs are easy and fast to build and apply to any study, independently of the specific resolution involved.

### 3.2. cDDMs Disclose Information about the Degree of Colocalization

The second analyzed dataset refers to a study of in vitro characterization of a drug delivery system, in which the authors verify the compartmentalization of the developed nanoparticles (PTX-Ce6@ker_ag_) into late endosomes (marked by Lamp-1 staining) [29] (Figure 3a, top).

We find that 19% of Ce6 signal overlaps with 17% of the Lamp-1 signal, with compatible MOC of 0.16 and ρ of 0.17. Such low MOC and ρ values are explainable by the small and sparse nature of the marked structures, which can also explain the low correlation values between markers’ local densities (Table 2, first and second columns).

However, the fact that the co-density-based refinement increases the correlation coefficients values while decreasing the area of investigation (and consequently M_1_ and M_2_’s value) hints at the capability of our method to selectively retain the colocalization between signals, more so than with false positives.

The cOM (Figure 3a) indicates the presence of signals overlap spots (Figure 3a, red spots in cOM magnification) enclosed in single-marker spots (Figure 3a, blue and yellow regions in cOM magnification), suggestive of NPs’ internalization into late endosomes. The cDDM (Figure 3a) further separates co-occurring pixels by cLDI, reporting co-densities dispersed across the cLDI range, with 11 out of 16 cLDIs capturing at least 5% of co-occurring pixels and only 13% of co-occurring pixels being also equally dense (i.e., cLDI = 0, Figure 3b). The similarity of all coefficients’ values between the first and second columns of Table 2 indicates the local density as being an indicator of colocalization, at least as valid as pixel intensity. Restricting correlation analysis to pixels with cLDI = 0 strongly increases ρ and ρ_s_ values (Table 2, third column), suggesting the existence of a real, although spatially limited, colocalization. Its detection by correlation coefficients is initially weakened by the scarcity of marked structures within the co-occurrence region, but subsequently strengthened by coDDM-driven increase in analysis specificity. Moreover, co-density analysis reveals that Ce6 signal tends to be locally denser than Lamp-1’s, as attested by the prevalence of negative values in the cDDM (Figure 3b). This last finding, in agreement with expectedly denser NPs due to their nanoformulation [28], also suggests that NPs’ internalization into late endosomes could occur at a ratio higher than 1:1, with many NPs entering the same endosomes at once. On one hand, this is positive for the pharmacokinetic-improving function of the developed system, but on the other, it opens up to the possibility that a different nanoformulation, producing less dense NPs, could result in better colocalization values and NPs internalization.

In summary, the local co-density analysis here improves colocalization quantification under different aspects. First, it advances the intensity correlation analysis, identifying the subregions where a stronger colocalization is likely to occur. Second, it provides indication about the degree of colocalization (here, the degree of internalization) that, in this case, is suggested to also occur at ratios different from 1:1. Finally, the cDDM also allows the formulation of new biological hypotheses, whose verification could lead to improvements in the developed drug delivery system.

### 3.3. cDDMs Open to the Formulation of New Biological Considerations

The third dataset analyzed concerns the assessment of co-distribution of axons, visualized by NF-200 immunostaining (red), and the surrounding myelin sheaths, visualized by Fluomyelin (green) in rat spinal cord (Figure 4a, top).

Both ascending and descending sensory and motor pathways run in the spinal cord and the quantitative evaluation of respective distribution in low-power micrographs would permit a rapid quantitative evaluation in physiological and pathological conditions, for example, after spinal cord injury. The cOM (Figure 4a) well presents a dorsoventral pattern, reasonably reflecting the distribution of sensory versus motor pathways. In fact, ascending sensory paths, localized in the dorsal funiculus and the external part of the lateral funiculus, reveal a different signals’ co-density compared to the other areas of the white matter, occupied by descending motor paths (Figure 4a, cOM, the two magnifications). Motor and sensory pathways are quite different under many aspects, such as axonal density, myelin sheaths thickness and percentage of unmyelinated fibers [34]. In particular, axonal density and myelin sheath thickness are lowered in sensory paths. The cDDM (Figure 4a) further investigates the co-occurrence region, in which the intensity correlation is quite fair but the overlap is suspiciously high (ρ *=* 0.55, MOC *=* 0.57, Table 3, first column).

Such a MOC value could be read as an artifact of the offsets that seem to characterize the FM signal (shifted up, scatterplot Figure 4b), that have been proved to positively affect the MOC, especially when a scarce correlation between the intensities is found [30]. In this sense, a less biased measure of co-occurrence can be derived from the cOM and the M_1_ and M_2_ coefficients. Most probably, these results Table 3, first and second columns) can be interpreted as an artifact of image resolution, which is not able to fully capture the concentric nature of the myelin signal, surrounding the axon, without overlapping. In any case, these results confirm the outcome of cDDM already seen in Lamp1-Ce6, where a reduction of the signals’ co-occurrence is coupled with a marked increase in correlation values (ρ and especially ρ_s_ value, Table 3, third column). In fact, the resolution problem seems to be alleviated by our approach, indeed reducing the signals’ overlap, quantified by M_1_ and M_2_ of about 40%. The increase in correlation coefficients also indicates that markers intensities should not be assumed a priori to linearly correlate, according to the functional heterogeneity of axons’ and myelin’s distribution in the tissue. Even though most of the co-occurring pixels are also equally dense (58%, cLDI = 0, Figure 4b), a remarkable prevalence of positive values in cDDM indicates axons’ tendency to be denser than myelin, agreeing with the reduced myelin sheaths thickness observable for some pathways. Indeed, a lower myelin thickness reasonably reflects a lower local density of FM, but not of NF200 signal, therefore bringing higher cLDI values and decorrelating the two markers’ density (Table 3, second column). Moreover, by locally analyzing cDDM, we can see that the local density pattern depends on the nature of the anatomical pathway (Figure 4a, cDDM left magnification), specifically being enriched in low values (hence, in myelin) in the proximity of the dorsal median sulcus (DMS) and in high values (hence, in less myelinated axons) away from it (Figure 4c, line plot of the pixel values underlying the red line in cDDM motor pathway magnification). In conclusion, in addition to also exemplifying its applicability at the tissue level, here, the cDDM provides new biological information, revealing and mapping the spatial heterogeneity of the myelination pattern, which could not be derived from the original image. This makes the local co-density an effective indicator of the local degree of myelination and the cDDM a possible discriminator of neuronal pathways.

### 3.4. GUI for cDDMs Creation

To allow any user to work with coDDMs, here, we introduce coDDMaker, an upgraded version of DDMaker, a software endowed with a user-friendly GUI, created with MATLAB*^®^* App Designer [26]. coDDMaker was conceived for the guided analysis of the distributions and co-distribution of marker pairs. Starting from RGB, greyscale or directly binary images and based on customed search window size, the software builds the markers’ DDMs, cDDM and cOM and tabulates their numerical content. With coDDMaker, we also introduce a module for the background correction of non-binary input images [35] and a module for their local segmentation to also be used as tools for image denoising. A detailed description of coDDMaker functionalities is provided in Appendix B. The software completes the colocalization analysis of a couple of images under standard setting (i.e., global image segmentation and WS *=* 3) in less than 30 *s* on entry-level computers, although the total elapsed time strongly depend on different factors (e.g., the size of the objects to be segmented), as exemplified in Supplementary Table S2. As much as DDMaker, or even more so, coDDMaker could serve as a checkpoint for long-lasting experiments, follow-up and large-scale studies, that can be monitored on-line and adjusted on the basis of the software feedbacks, therefore, optimizing time and costs. coDDMaker is available as a public open-source software written in MATLAB*^®^* and as a 64-bit stand-alone application (https://sourceforge.net/projects/coddmaker/ accessed on 10 September 2021).

## 4. Conclusions

Image colocalization is commonly assessed by a combination of co-occurrence and correlation. However, all current methods exploiting pixel intensities neglect information regarding pixel interconnections that, if considered, could permit the enforcement of colocalization information. In this perspective, we introduce the co-density distribution map, a novel tool for improving the actual colocalization analysis framework in biomedical images. Given two imaged markers and, having built their density distribution maps, the cDDM uniquely describes the distribution of the signals’ local densities, in terms of relative position and abundance of marked structures. When imaging objects above the pixel resolution, the cDDM also becomes a powerful indicator of colocalization, which can identify the image regions at which colocalization is stronger, adding reliability to the correlation coefficients normally employed. The cDDM also provides information about the degree of colocalization, which can complement and validate quantitation by other methods. Most importantly, cDDM’s information is, altogether, qualitative, quantitative and local, making it a powerful tool for the fast and comprehensive surveyance of imaged systems. Consequently, it can open the door to new biological considerations, both at the global and the regional level. Working locally, DDMs (and cDDMs consequently) can increase the confidence of colocalization when this is not achievable by increasing the acquisition resolution, thus enhancing the information regarding distributions. Notably, our maps can be applied to any resolution study. In addition, being easy to build, the cDDM can benefit routine, large-scale and follow-up experiments by providing a tool for near real-time monitoring to be used for the adjustment and optimization of experiments. In practice, the cDDM we propose represents a fundamental tool to be integrated into each colocalization analysis framework, whether it is based on intensity correlation or not, to be used synergically with correlation analysis by masking the original images before computing the different coefficients. Even though it provides only an indication and not a direct measure of the degree of colocalization and, at present, it only works for the colocalization of two signals, the cDDM can be used to answer a variety of biological questions involving protein–protein interactions or co-compartmentalization. As a future research direction, we are working on a stand-alone tool capable of providing a new indicator of colocalization merging the information from pixel intensity and density.

## Figures and Tables

**Table 1:** Comparison between binary erosion and co-density analysis in refining the correlation computation domain.

MASKS		NF200-FM		SYP-VGLUT1		Lamp1-Ce6	
Co-occurrence(before refinement)	Pixel nr (% ^1^)	1465036	(100)	9343	(100)	737	(100)
	Object nr (%)	19068	(100)	968	(100)	199	(100)
	ρ (ρ_s_)	0.5535	(0.3760)	0.2406	(0.1286)	0.1666	(0.1656)
Binary erosion refinement (*4-conn*) ^2^	Pixel nr (%)	957332	(65.35)	3011	(32.23)	88	(11.94)
	Object nr (%)ρ (ρ_s_)	11244	(58.97)	244	(25.21)	24	(12.06)
		0.6170	(0.4456)	0.3353	(0.2112)	0.1479	(0.1459)
Binary erosion refinement (8*-conn*) ^2^	Pixel nr (%)	810579	(55.33)	1865	(19.96)	31	(4.21)
	Object nr (%)	10162	(53.29)	158	(16.32)	9	(4.52)
	ρ (ρ_s_)	0.6416	(0.4736)	0.3707	(0.2536)	0.3454	(0.3288)
cDDM refinement ^3^	Pixel nr (%)	851042	(58.09)	2394	(25.62)	99	(13.43)
	Object nr (%)	16300	(85.48)	378	(39.05)	46	(23.12)
	ρ (ρ_s_)	0.6508	(0.5031)	0.4824	(0.4635)	0.5156	(0.4353)

^1^ Percentages refer to the co-occurrence region (pixel or object number) before its refinement. ^2^ 3 × 3 structuring element. ^3^ 3 × 3 search window.

**Table 2:** Comparison between Lamp-1 and PTX-Ce6@ker_ag_ intensity and local density colocalization analysis, before and after refinement for local co-density.

	Lamp1-Ce6		
	Co-Occurrence Region (n * = 737)		Co-Density Region (n * = 99)
	Intensity	Density	Intensity
ρ	0.1666	0.1278	0.5156
ρ_s_	0.1656	0.1270	0.4353
MOC	0.1564	0.1669	0.9059
M_1_	0.1852	0.1662	0.0246
M_2_	0.1712	0.1958	0.0275

* n: sample size, or number of considered pixels.

**Table 3:** Comparison between NF200 and FM intensity and local density colocalization analysis, before and after refinement for local co-density.

	NF200-FM		
	Co-Occurrence Region (n * = 1,465,036)		Co-Density Region (n * = 851,042)
	Intensity	Density	Intensity
ρ	0.5535	0.2064	0.6508
ρ_s_	0.3760	0.2520	0.5031
MOC	0.5741	0.7221	0.9782
M_1_	0.4909	0.5060	0.2983
M_2_	0.6772	0.6601	0.4212

* n: sample size, or number of considered co-occurring pixels.

**Figure A1:**
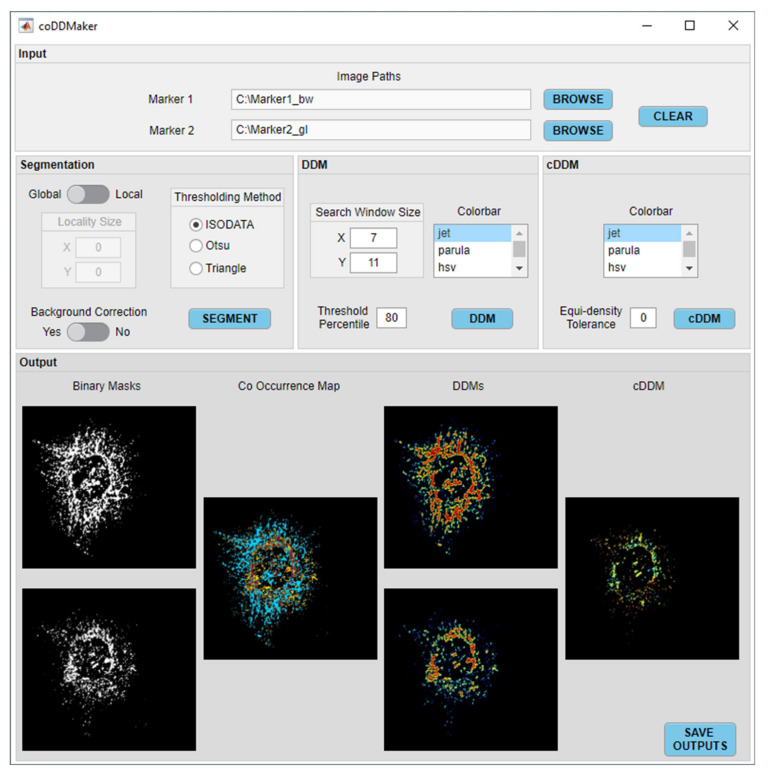
Main GUI of *coDDMaker*. The main window is divided into five sections: (1) Input: to select the input images’ folders; (2) Segmentation: to select the thresholding method, its locality of application and to eventually perform background correction before threshold calculation and image binarization; (3) DDM: to select the size of the search window for local density analysis and, to allow user creating and binarizing DDMs after setting the colorbar for pseudo-color DDMs visualization and the percentile for DDMs thresholding; (4) cDDM: to allow user creating and binarizing cDDMs after setting, the colorbar for pseudo-color cDDMs visualization and the tolerance for equi-density region segmentation; (5) Output: to visualize and save intermediates and outputs. From left to right: markers binary masks, cOM, pseudo-color DDMs, pseudo-color cDDM.

## Data Availability

No new data were created or analyzed in this study. Data sharing is not applicable to this article.

## Supplementary Materials

The following are available online at https://www.mdpi.com/article/10.3390/s21196385/s1, Table S1: Comparison between SYP and VGLUT1 intensity and local density colocalization analysis, before and after refinement for local co-density, Figure S1A: Scatterplots of co-occurrence region partitioning by equi-density, Figure S1B: Scatterplots of co-occurrence region partitioning by cLDI sign, Figure S1C: Scatterplots of co-occurrence region partitioning by cLDI value, Table S2: coDDMaker time performance evaluation, Table S3: Abbreviations.

## Appendix A

### Appendix A.1. Correlation and Co-Occurrence Coefficients

#### Appendix A.1.1. Pearson’s Correlation Coefficient

The Pearson’s correlation coefficient (ρ, r, or PCC), is defined as the quotient of covariance and standard deviation between two variables:

(A1) $$\rho =\frac{\sum_{i}^{n}\left(x_{i}-\;x¯\right)*\sum_{i}^{n}\left(y_{i}-\;y\text{¯}\right)}{\sqrt{\sum_{i}^{n}{\left(x_{i}-\;x¯\right)}^{2}*\sum_{i}^{n}{\left(y_{i}-\;y\text{¯}\right)}^{2}}}$$

When these two variables (x and y) describe the pixel intensity of two probes’ signals imaged in the same domain (i.e., the signals’ co-occurrence region, composed of n pixels), Equation (A1) can be used to quantify the extent of linear association between the signals intensities, as a measure of probes colocalization [6,21]. Many reviews discuss the coefficient history [36], meaning [15,36] and implications for image colocalization [4,8,13,24,30].

### Appendix A.1.2. Spearman’s Correlation Coefficient

The Spearman correlation coefficient (ρ_s_, r_s_ or SRCC) is defined as the Pearson’s correlation coefficient between the rank variables [29] and it is then computed simply by replacing x and y intensity values with intensity ranks of values in Equation (A1). By working on ranks, ρ_s_ assesses how well the relationship between two variables can be described using a monotonic function, disregarding any assumption of linearity [37].

### Appendix A.1.3. Mander’s Coefficients

In 1993 Manders introduced the overlap coefficient to supply the lack of interpretability for Pearson’s coefficients negative values [6]:

(A2)
$$\mathrm{MOC}=\frac{\sum_{i}^{n}x_{i}*\sum_{i}^{n}y_{i}}{\sqrt{\sum_{i}^{n}{\left(x_{i}\right)}^{2}*\sum_{i}^{n}{\left(y_{i}\right)}^{2}}}$$

By simply removing the average subtraction from the intensity values, the coefficient is claimed to become “insensitive to differences in signal intensities between the components of an imaged caused by different labelling with fluorochromes, photo-bleaching, or different settings of the amplifiers”. However, to increase the coefficient interpretability, Manders further introduces the M_1_ and M_2_ coefficients:

(A3)
$$M_{1}=\frac{\sum_{i}^{n}x_{i,\mathrm{coloc}}}{\sum_{i}^{n}x_{i}}\;\;\left\{\begin{array}{c} x_{i,\mathrm{coloc}}=x_{i},ify_{i}>0\\ x_{i,\mathrm{coloc}}=0,ify_{i}=0 \end{array}\right.$$

(A4)
$$M_{2}=\frac{\sum_{i}^{n}y_{i,\mathrm{coloc}}}{\sum_{i}^{n}y_{i}}\;\;\;\left\{\begin{array}{c} y_{i,\mathrm{coloc}}=y_{i},ifx_{i}>0\\ y_{i,\mathrm{coloc}}=0,ifx_{i}=0 \end{array}\right.$$

M_1_ and M_2_ separate the fluorophores contribution to colocalization, by calculating for each fluorophore the fraction of the total intensity that co-occurs.

Manders’ M_1_ and M_2_ coefficients have since then been widely used to quantify signals co-occurrence in intensity images. However, doubts on the suitability of the MOC coefficient to the quantification of unbiased co-occurrence have been casted [30] and its use along with M_1_ and M_2_ coefficients is currently a topic under heavy discussion [4,31,32,33].

## Appendix B

### coDDMaker: GUI Description

To enable users to build customizable DDMs and cDDMs, we realized *coDDMaker* (Figure A1), a software program endowed with a user-friendly GUI created with MATLAB^®^ App Designer.

The software was conceived as an upgrade of the *DDMaker* software [26] and permits the performance of a density-including colocalization analysis of two markers’ co-distribution. As before, the software does not require any training or expertise before use. Preserving the original design, we added new modules for: (1) background correction for uneven illumination [35], (2) local image segmentation, (3) cDDM and cOM building and (4) saving of all numerical and visual outputs of the analysis for further investigation.

First, the user is required to select the two folders (i.e., one for each marker to be analyzed) where the input images to be processed are located. Images can either be RGB color, grey level or binary, in the MATLAB-supported formats [38]. The user can binarize RGB and grey level images by choosing among ISODATA or Otsu thresholding method. With coDDMaker, the user can now also decide to apply the thresholding algorithm locally, by specifying the locality dimension. The Triangle method is also supplied for global image thresholding, where it can serve outliers removal in heavy-tailed histograms. In addition, images can now be pre-processed for the correction of uneven illumination that may result from vignetting distortion, inaccurate image acquisition or noise [35]. Binary masks, resulting from thresholding or already provided by the user, serve as the input for building the DDMs, cDDM and cOM. Local density analysis is performed using a default search window of 3 × 3 pixels, chosen assuming that the target structures of interest in the images are of few pixels, thus enabling the detection of small aggregation events and single particles as well. However, users can customize the search window size, besides the color bar for maps visualization. The resulting DDMs can be binarized by percentile thresholding, while the equi-density region, identified in the cDDM by cLDI = 0, can be binarized by setting a co-density tolerance (e.g., a tolerance of two identifies as co-dense pixels with cLDI ranging from 0 − 2 to 0 + 2). To help the user in finding the best parameter setting for its analysis, coDDMaker also displays the last binary mask for each marker and the derived DDMs, cDDM and cOM. When satisfied with the setting, the user can save all the intermediate and final outputs of the analysis, which include all the generated images and maps and the numerical data associated to DDMs and cDDMs. The images are saved in uncompressed TIFF format, while other analyses’ outputs are saved as portable csv and excel files. A detailed explanation of coDDMaker utilization can be found in the software documentation (https://sourceforge.net/projects/coddmaker/ accessed on 10 September 2021).
