# Supplementary material for: Co-Density Distribution Maps for Advanced Molecule Colocalization and Co-Distribution Analysis
Source: Sensors (Basel). 2021 Sep 24;21(19):6385. doi: 10.3390/s21196385 (PMC8513075; doi:10.3390/s21196385)
Supplement: Supplementary file 1 [file sensors-21-06385-s001.zip › sensors-1338078-supplementary.pdf]

sensors

Supplementary Materials

Co-Density Distribution Maps for Advanced Molecule Colocalization and Co-Distribution Analysis

Ilaria De Santis [1,2], Luca Lorenzini [3], Marzia Moretti [4], Elisa Martella [5], Enrico Lucarelli [6], Laura Calzà [4,7] and Alessandro Bevilacqua [8,9,*]

1 Department of Medical and Surgical Sciences (DIMEC), Alma Mater Studiorum—University of Bologna, I-40138 Bologna, Italy; i.desantis@unibo.it
2 Interdepartmental Center Alma Mater Research Institute on Global Challenges and Climate Change (Alma Climate), Alma Mater Studiorum—University of Bologna, I-40126 Bologna, Italy
3 Department of Veterinary Medical Sciences (DIMEVET), Alma Mater Studiorum—University of Bologna, I-40064 Ozzano Emilia, Italy; luca.lorenzini8@unibo.it
4 Iret Foundation, I-40064 Ozzano Emilia, Italy; marzia.moretti3@unibo.it (M.M.); laura.calza@unibo.it (L.C.)
5 Institute of Organic Synthesis and Photoreactivity (ISOF), National Research Council (CNR), I-40129 Bologna, Italy; elisa.martella@isof.cnr.it
6 Regenerative Therapies in Oncology, IRCCS Istituto Ortopedico Rizzoli, I-40136 Bologna, Italy; enrico.lucarelli@ior.it
7 Department of Pharmacy and BioTechnology (FaBiT), Alma Mater Studiorum—University of Bologna, I-40127 Bologna, Italy
8 Advanced Research Center on Electronic Systems (ARCES) for Information and Communication Technologies "E. De Castro", Alma Mater Studiorum—University of Bologna, I-40125 Bologna, Italy
9 Department of Computer Science and Engineering (DISI), Alma Mater Studiorum—University of Bologna, I-40136 Bologna, Italy
* Correspondence: alessandro.bevilacqua@unibo.it; Tel: +39-051-2095409

Citation: De Santis, I.; Lorenzini, L.; Moretti, M.; Martella, E.; Lucarelli, E.; Calzà, L.; Bevilacqua, A. Co-Density Distribution Maps for Advanced Molecule Colocalization and Co-Distribution Analysis. *Sensors* **2021**, *21*, 6385. https://doi.org/10.3390/s21196385

Academic Editors: Sheryl Berlin Brahnam, Loris Nanni, Rick Brattin

Received: 28 July 2021
Accepted: 21 September 2021
Published: 24 September 2021

Publisher's Note: MDPI stays neutral with regard to jurisdictional claims in published maps and institutional affiliations.

Copyright: © 2021 by the authors. Licensee MDPI, Basel, Switzerland. This article is an open access article distributed under the terms and conditions of the Creative Commons Attribution (CC BY) license (http://creativecommons.org/licenses/by/4.0/).

Table S1. Comparison between SYP and VGLUT1 intensity and local density colocalization analysis, before and after refinement for local co-density.

	SYP-VGLUT1		
	**co-occurrence region (n*=9343)**		**co-density region (n*=2394)**
	**intensity**	**density**	**intensity**
ρ	0.2406	0.0773	0.4824
ρ_s	0.1286	0.0953	0.4635
MOC	0.2420	0.2325	0.9317
M_1	0.1386	0.1354	0.0411
M_2	0.4574	0.4530	0.1479

* n: sample size, or number of considered co-occurring pixels.

Figure S1A. Scatterplots of co-occurrence region partitioning by equi-density.

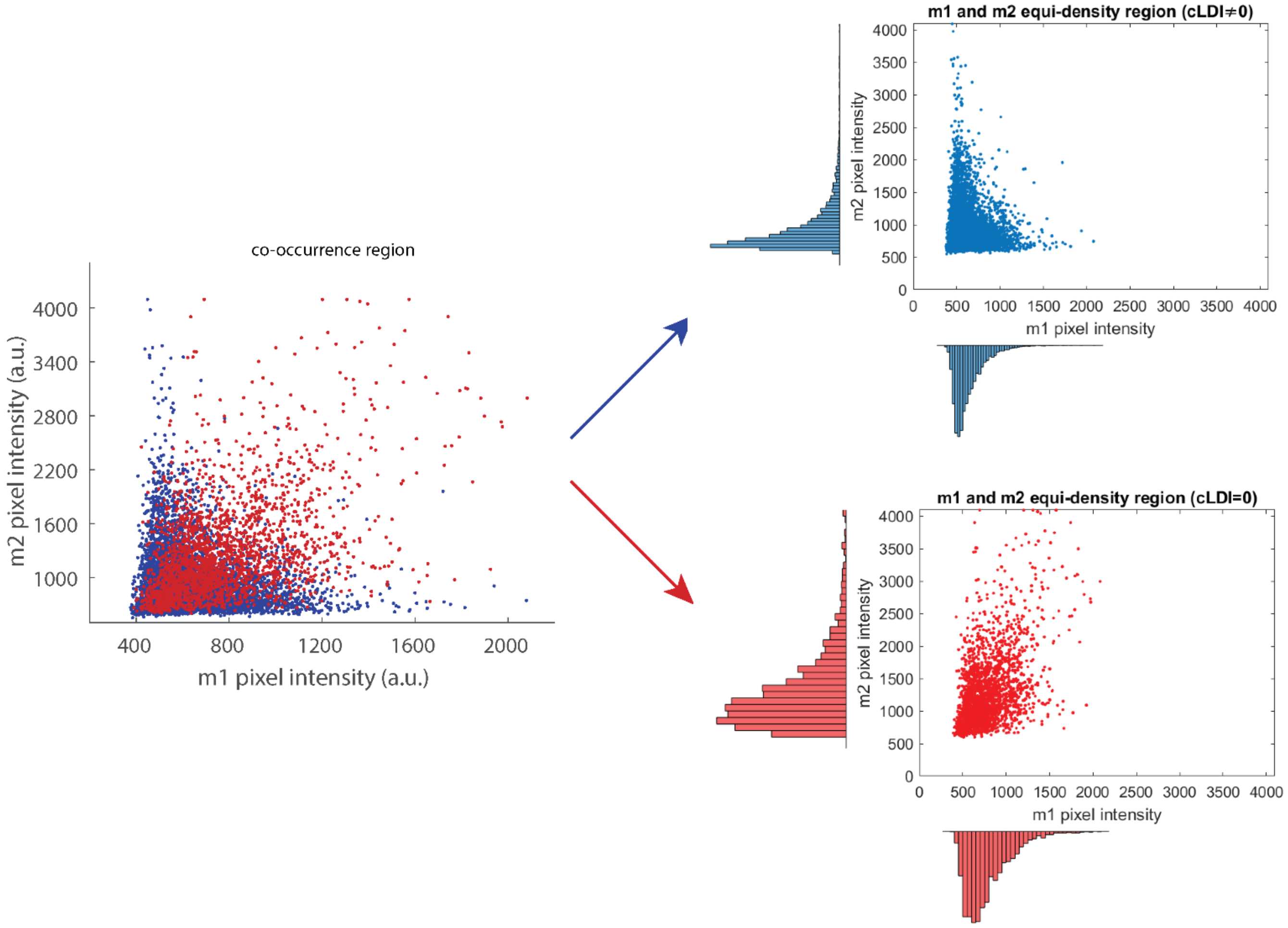

Figure S1B. Scatterplots of co-occurrence region partitioning by cLDI sign.

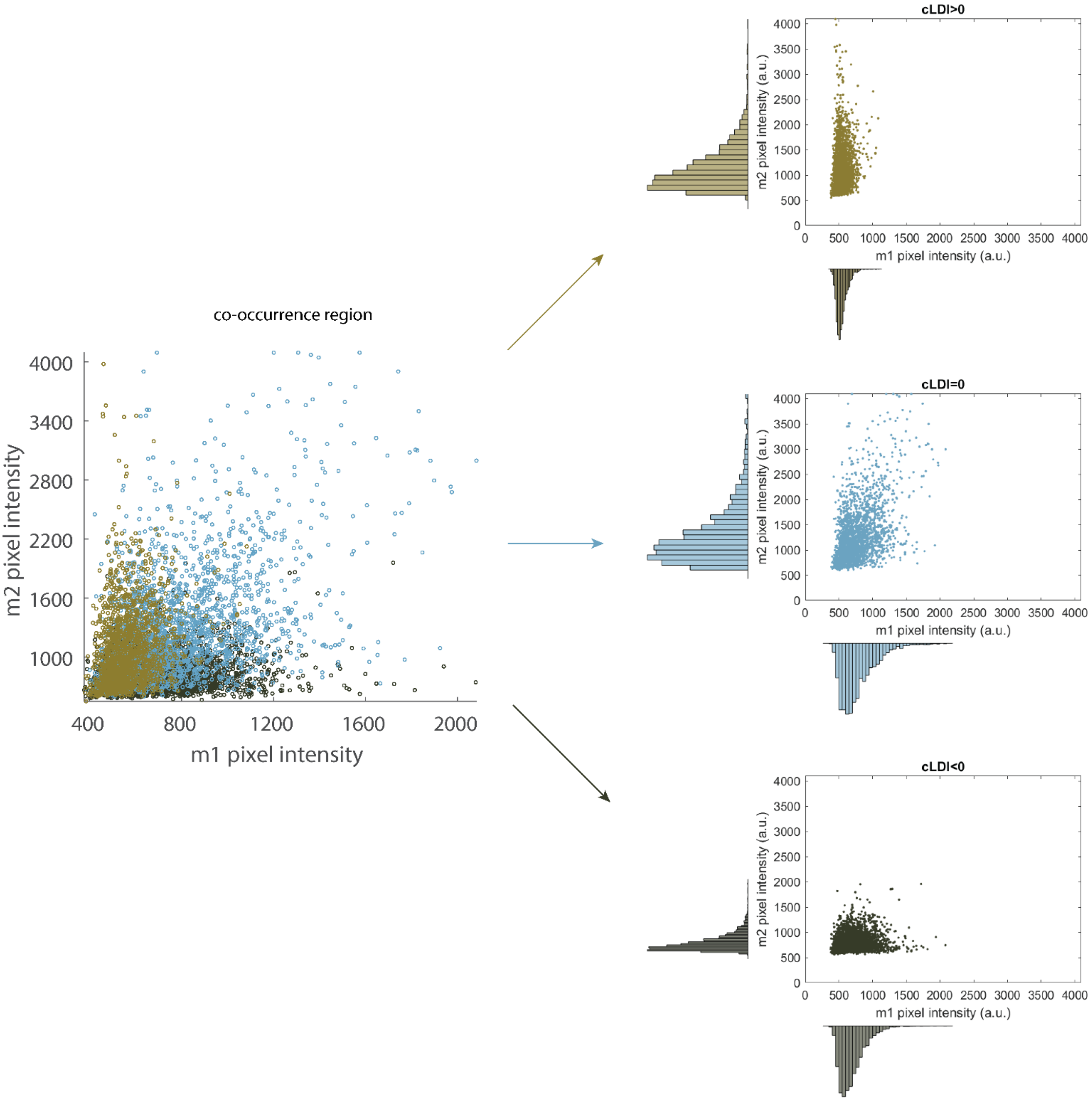

Figure S1C. Scatterplots of co-occurrence region partitioning by cLDI value.

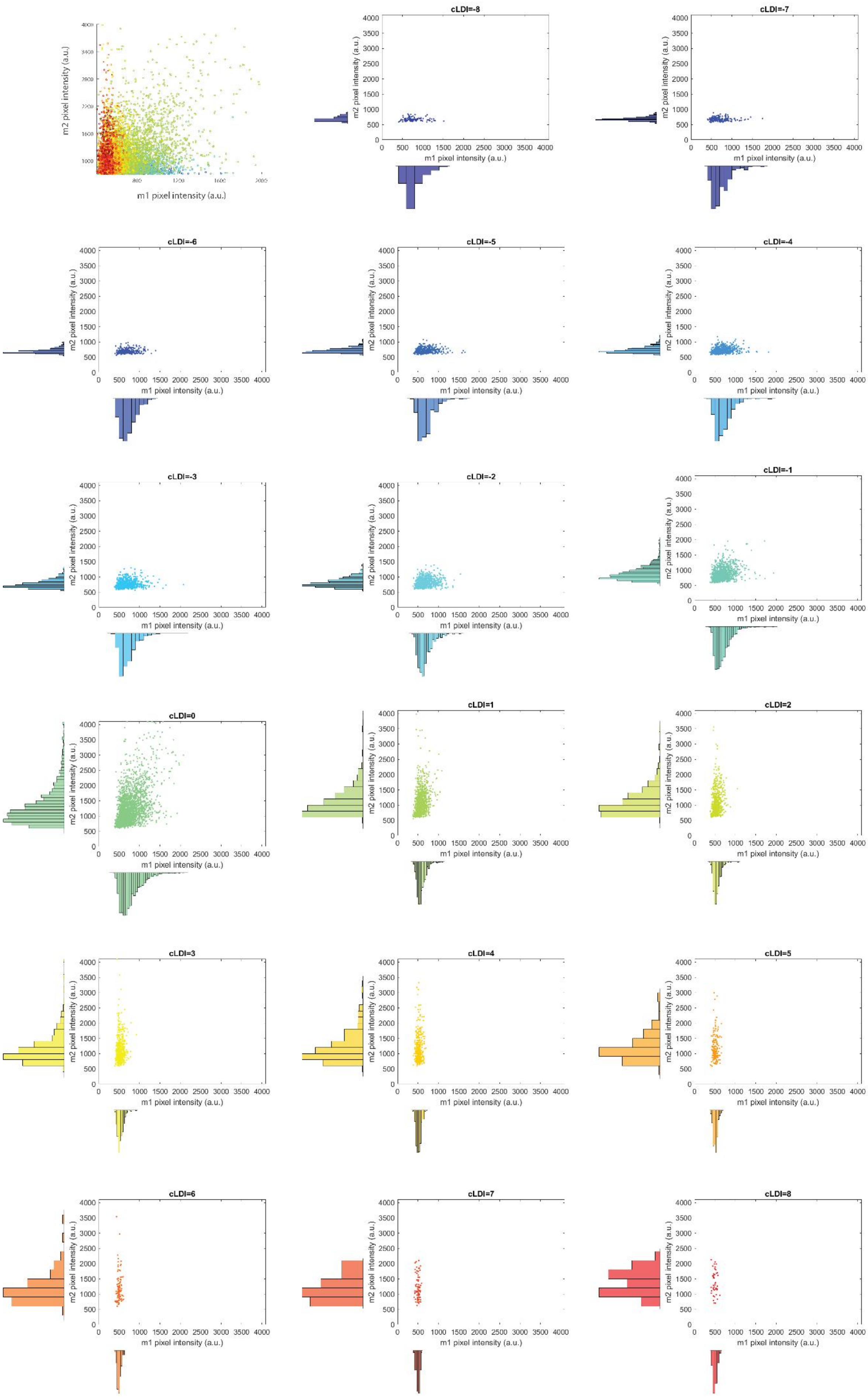
m2 pixel intensity (a.u.)
m1 pixel intensity (a.u.)
cLDI=-8
cLDI=-7
cLDI=-6
cLDI=-5
cLDI=-4
cLDI=-3
cLDI=-2
cLDI=-1
cLDI=0
cLDI=1
cLDI=2
cLDI=3
cLDI=4
cLDI=5
cLDI=6
cLDI=7
cLDI=8

Table S2. *coDDMaker* time performance evaluation.

Image size	Background correction	Locality size (image segmentation)[1]	Search window size (density analysis)	Elapsed Time[2]
1024×1024	No	Global	3×3	20″
512×512	No	Global	3×3	15″
512×512	Yes	Global	3×3	20″
512×512	Yes	3×3	3×3	50″
512×512	Yes	15×15	3×3	1′20″
512×512	Yes	15×15	9×9	1′25″

[1]Otsu thresholding method. [2]Local density and co-density analysis in a couple images on a PC endowed with Intel i3-4005U, 1.70 GHz processor, and 8 GB RAM.

Table S3. Abbreviations.

**BW**	Black and White image (binary mask)
**cDDM**	Co-Density Distribution Map
**cLDI**	Local co-Density Index
**cOM**	Co-Occurrence Map
**DDM**	Density Distribution Map
**DMS**	Dorsal Median Sulcus
**FG**	Foreground
**FM**	Fluoromyelin®
**GUI**	Graphical User Interface
**HPL**	Helix Pomatia Lectin
**IF**	Immunofluorescence
**Lamp-1**	Lysosomal-Associated Membrane Protein 1
**LDI**	Local Density Index
**M1**	Mander's coefficient 1
**M2**	Mander's coefficient 2
**MOC**	Manders' Overlap Coefficient
**NF**	Neurofilament
**PTX**	Paclitaxel
**SYP**	Synaptophysin
**VGLUT1**	Vesicular Glutamate Transporter 1
**WS**	(Search) Window Size
ϱ	Pearson's correlation coefficient (also r, or PCC)
ϱ_s	Spearman's correlation coefficient (also r_s, or SRCC)
